# An Intact Centrosome Is Required for the Maintenance of Polarization during Directional Cell Migration

**DOI:** 10.1371/journal.pone.0015462

**Published:** 2010-12-23

**Authors:** Nicole M. Wakida, Elliot L. Botvinick, Justin Lin, Michael W. Berns

**Affiliations:** 1 Beckman Laser Institute, University of California Irvine, Irvine, California, United States of America; 2 Developmental and Cell Biology, University of California Irvine, Irvine, California, United States of America; 3 Department of Biomedical Engineering, University of California Irvine, Irvine, California, United States of America; UCLA and Cedars-Sinai Medical Center, United States of America

## Abstract

**Background:**

Establishing and maintaining polarization is critical during cell migration. It is known that the centrosome contains numerous proteins whose roles of organizing the microtubule network range include nucleation, stabilization and severing. It is not known whether the centrosome is necessary to maintain polarization. Due to its role as the microtubule organizing center, we hypothesize that the centrosome is necessary to maintain polarization in a migrating cell. Although there have been implications of its role in cell migration, there is no direct study of the centrosome's role in maintaining polarization. In this study we ablate the centrosome by intracellular laser irradiation to understand the role of the centrosome in two vastly different cell types, human osteosarcoma (U2OS) and rat kangaroo kidney epithelial cells (PtK). The PtK cell line has been extensively used as a model for cytoskeletal dynamics during cell migration. The U2OS cell line serves as a model for a complex, single migrating cell.

**Methodology/Principal Findings:**

In this study we use femtosecond near-infrared laser irradiation to remove the centrosome in migrating U2OS and PtK2 cells. Immunofluorescence staining for centrosomal markers verified successful irradiation with 94% success. A loss of cell polarization is observed between 30 and 90 minutes following removal of the centrosome. Changes in cell shape are correlated with modifications in microtubule and actin organization. Changes in cell morphology and microtubule organization were quantified revealing significant depolarization resulting from centrosome irradiation.

**Conclusions/Significance:**

This study demonstrates that the centrosome is necessary for the maintenance of polarization during directed cell migration in two widely different cell types. Removal of the centrosome from a polarized cell results in the reorganization of the microtubule network into a symmetric non-polarized phenotype. These results demonstrate that the centrosome plays a critical role in the maintenance of cytoskeletal asymmetry during cell migration.

## Introduction

Cell migration is a complex phenomenon requiring the reorganization of numerous components and organelles into a polarized state. Asymmetric positioning of the various cellular components promotes turnover and movement of necessary signaling, cytoskeletal, and membranous elements. Classically, a polarized cell has been defined by the positions of the actin-rich lamellae, centrosome, and Golgi apparatus between the lamellae and posterior-positioned nucleus. In addition, microtubules concentrate and stabilize within the lamella, allowing for vesicular transport to the leading edge of the cell [1]. The contribution of the actin network has been the focus of most cell migration studies and serves as the protrusion force of the lamellae via polymerization [2] as well as controlling spreading and contraction of the tail in concert with focal adhesions [3].

Recent advances have given us a better understanding of the role of microtubules in cell migration. The first study to demonstrate that microtubules were involved in directed cell migration was reported in 1970 [4]. Here the authors demonstrated that directional migration of mouse and human embryonic fibroblast-like cells were inhibited by the addition of the microtubule destabilizing drug colcemid. More recently it has been shown that the organization of cellular architecture, including the position of the Golgi apparatus, is dependent on an intact microtubule cytoskeleton [5]. Recent studies have shown that microtubules have multiple roles in the migration process including polarization of signaling molecules [3], maintenance of cell shape [6] and dissociation of adhesion sites [3]. Specifically, it has been shown that microtubules mediate changes in Rho GTPase activity at sites of substrate adhesion to promote adhesion disassembly and remodeling of the actin cytoskeleton [7], [8].

Early studies suggested the requirement of microtubules for directed cell migration is cell type dependent. In 1984, Euteneur and Schliwa [9] had reported that fast migrating cells including keratocytes and neutrophils can directionally migrate in the absence of microtubules. In contrast, recent studies suggest that disruption of the microtubule network in T cells reduces the rate of migration and are subject to frequent directional changes due to the use of membrane blebbing based migration. Thus the microtubule network is required for persistent polarization and optimal migration in T cells [10].

As the primary organizing center of microtubules, it would be logical that the centrosome plays a vital role in cell migration. The centrosome is composed of numerous proteins responsible for microtubule nucleation, anchoring, and release [11]. The role of the centrosome is very complex as suggested by studies showing that the position of the centrosome can vary depending on conditions of migration within the same cell type [12], and between cell types [13]. Evidence of the centrosome's role in migration stems from a study focusing on cell migration in *Dictyostelium discoideum* which found that the pseudopod extends for an average 12 seconds before centrosome reorientation, and if the centrosome did not reorient within 30 seconds, the pseudopod retracted [14]. This study suggests that the repositioning of the centrosome stabilizes the direction of movement through the microtubule system. As early as 1979, studies have shown the preferential orientation of centrioles perpendicular and parallel to the substrate suggesting the involvement of centrioles in controlling cell migration [15].

Laser microirradiation was first used in 1984 to provide experimental evidence that centrioles played a role in cell migration [16]. In this study, a UV laser microbeam irradiated a granule free zone in migrating new eosinophils that corresponded to the position of the centrioles in the cell. Laser irradiation of this region resulted in a 50% reduction in migration rate, and loss of directed cell migration. Subsequently, advances in molecular and microscopy techniques have allowed for more precise determination of the position of the centrosome in the cell. The combination of GFP-fusion proteins that label the centrioles and/or the surrounding pericentriolar material (PCM) of the centrosome and the availability of primary antibodies for centrosome proteins permit accurate targeting of the centrosome and rapid determination of successful irradiation events [17].

One of the advantages of laser irradiation is the instantaneous (femtosecond to nanosecond) change to the irradiated area without additional deleterious effects to the rest of the targeted cell. Studies using electron microscopy [18], [19] and immunofluorescence [20] have demonstrated the use of laser microirradiation to remove the centrosome and successfully observe subsequent cell behavior in the irradiated as well as daughter cells [21].

In this study we demonstrate in human osteosarcoma (U2OS) and rat kangaroo kidney epithelial cells (PtK2), that ablation of the centrosome results in the loss of the cell's ability to maintain a polarized microtubule network necessary for cell migration in a specific direction. We present the first direct experimental evidence of the centrosome's connection to cell polarization. Our findings suggest the centrosome is necessary to maintain cytoskeletal and overall cell polarization during migration in multiple cell types, suggesting a new role for the centrosome during cell migration.

## Results

### Irradiation of the centrosome

**Figure 1:**
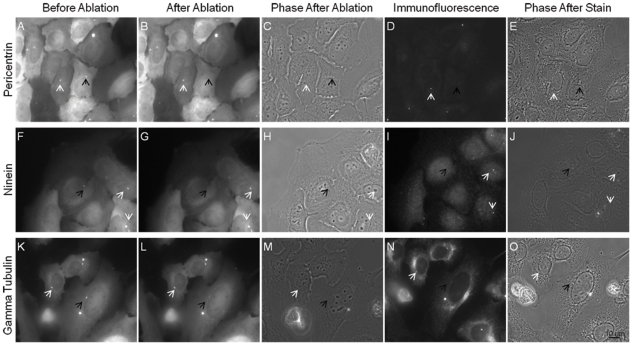
Immunofluorescence staining with percentrin, ninein, and gamma tubulin of centrosome irradiated cells. The laser was exposed to the centrosome region as identified by the presence GFP fluorescence in A,F,K. Following laser exposure a successful irradiation event was determined by loss of fluorescence or fragmentation of the centrosomal marker signal at the position of irradiation (black arrow). Three different centrosomal probes were used where 27 of 28 pericentrin stained cells, 22 of 24 ninein stained cells, and 19 of 20 gamma tubulin stained cells were determined successful. Both GFP and immunofluorescence signal of centrosomes in neighboring control cells were not affected following laser exposure as pointed out by the white arrows in B, G, and L. Phase images after irradiation (C,H, and M) were matched following the staining procedure (E, J, and O).

A femtosecond laser irradiation system [22] was used to target the GFP-labeled centrin region in single migrating U2OS cells. Fluorescence images using GFP-labeled centrin acquired before laser exposure (Figure 1A,F,K), immediately after laser exposure (Figure 1B,G,L), and corresponding phase images after laser exposure are presented (Figure 1C,H,M). A loss of fluorescence is observed at the irradiated “Region Of Interest” (ROI) immediately following laser exposure (black arrow) indicative of either photobleaching or molecular destruction. We determined the laser parameters for molecular destruction as shown in Figure 1D,I,N verified by post ablation immunofluorescence. GFP signals from control non-irradiated centrosomes in neighboring cells were not affected by the laser exposure (white arrows).

### Immunofluorescence staining of irradiated cells

Immunofluorescence staining was carried out on cells fixed within 1–30 minutes following laser exposure. Cells were stained using multiple centrosomal markers including pericentrin, ninein, and gamma tubulin (Figure 1D,I,N). In 94% of irradiated cells a loss of GFP signal following laser exposure corresponded to an absence of staining for centrosomal markers at the same position. This percentage was based on the loss of immunofluorescence signal for 27 of 28 pericentrin stained cells, 22 of 24 ninein stained cells, and 19 of 20 gamma tubulin stained cells. Neighboring centrosomes (white arrows, Figure 1) were not affected by the laser exposure and exhibited normal GFP fluorescence for all three centrosomal markers.

### Laser irradiation affect on cell shape

We first determined if non-centrosome targeted laser exposure had an effect on cell polarization. A random position in the cytoplasm not containing the centrosome in migrating cells was exposed to the laser. Figure S1 illustrates the progression of four control-irradiated cells followed for 90 or more minutes post cytoplasmic irradiation. Laser power settings and size of the irradiated ROI for the control-irradiated cells were identical to those utilized for centrosome irradiation (see white-outlined ROI in Figure S1H). Throughout the observation period cells remained mobile, changing their shape but retaining their polarized morphology. All cells maintained a distinct but dynamic lamellae structure, often sending out filopodia (Figure S1F,I). Ruffling of the membrane was observed throughout the observation period (Movie S1 and S2) for both the cytoplasmic and centrosome irradiated cells.

**Figure 2:**
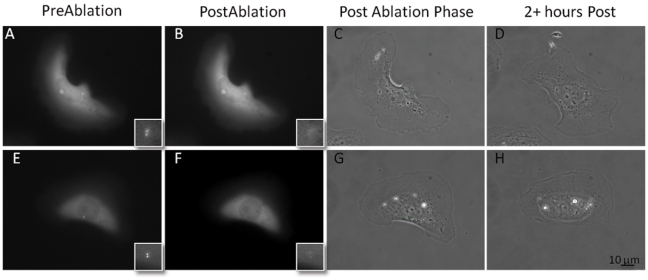
Centrosome irradiation causes a distinct change in cell morphology over a 2 hour period. A,E show GFP fluorescence images of cell prior to irradiation. B,F show fluorescence images of cell immediately following irradiation. The inset represents a magnified image of the centrosome region exposed to the laser. Loss of fluorescence at these positions following laser exposure is evident in magnified insets. C,G show phase contrast images of cell immediately following laser irradiation. Cells do not appear to be affected by laser exposure. D,H show the phase contrast images of cells observed for 2 or more hours following centrosome irradiation. In 22 of 26 centrosome irradiated cells, morphology appears less polarized when compared to images taken immediately following irradiation. Scale bar  = 10 µm.

Laser irradiation of the centrosome in single cells resulted in changes in cell morphology that persisted for two or more hours following irradiation (Movie S1). In figure 2 the pre and post-irradiation observation of two U2OS cells selected for centrosome irradiation are presented. Prior to laser irradiation, each cell possesses 2–4 fluorescent points that correspond to the centrioles within the centrosome (Figure 2A,E). Immediately following laser exposure, a loss of fluorescence was observed at the targeted position indicating a successful centrosome deletion event (Figure 2B,F). The green box surrounding the fluorescent centrosome corresponds to the region exposed to the laser. No significant changes were observed between phase contrast images before and immediately following centrosome irradiation (Figure 2C,G) suggesting that there no gross ablation event as would be observed if the laser irradiation induced a micro-plasma-generated cavitation and mechanical shock waves [18]. In 22 of 26 centrosome irradiated cells, a loss of the cell's polarized morphology was observed in phase contrast images acquired two or more hours following irradiation (Figure 2D,H). Cells changed to a more symmetric shape following the loss of a distinct lamellae. Centrosome irradiated cells appeared more rectangular (Figure 2D) or circular in shape (Figure 2H) with a more symmetrical organization of the cytoplasm with respect to the nucleus as compared to control cells. Centrosome deleted cells did not reestablish a polarized morphology during the observation period (up to 20 hours following laser irradiation).

**Figure 3:**
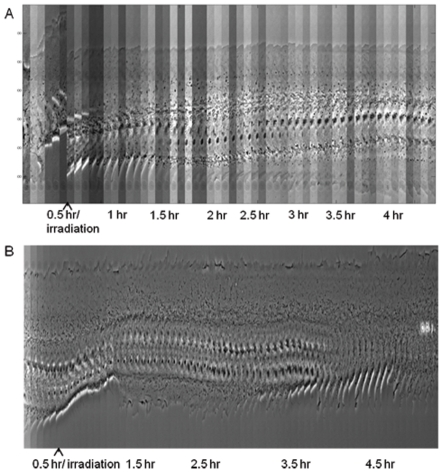
Kymograph of a centrosome irradiated cell and cytoplasm irradiated cell. (A) Initially we observe forward movement of both the leading edge (top) and trailing edge (bottom) as determined by the incline formed by the cell edges. After the first 30 minute period, the cell was repositioned and the centrosome was irradiated by the laser. Following laser exposure we see no forward movement of the leading edge as determined by the horizontal line formed by the leading edge (top). At the trailing edge (bottom) we see a spreading of the cell edge forming a declining line. By the end of the 4.5 hour observation period, the cell front and back appear similar to each other and equally spaced from the nucleus. See Movie S1. (B) Over time, we see the cell constantly changing its shape and position. Regardless of its shape change, there is always a visible lamellae with a majority of the cytoplasm in front of the nucleus (in the direction of migration). See Movie S2.

To better understand the changes in cell morphology, kymographs were constructed for both a centrosome (Figure 3A) and cytoplasm (Figure 3B) irradiated cell. The cells were stimulated with BMP-2 (give concentration) which results in directed forward migration. During the initial 30 minutes following BMP-2-stimulation, both the front and the back of the cell form an ascending line in the kymograph, depicting the forward movement of the cell. At 30 minutes the cells were repositioned and either the centrosome or a region in the cytoplasm (control cells) was irradiated, corresponding to the vertical shift of the cell in the kymograph. Immediately following irradiation of the centrosome, cessation in the progression of the leading edge of the cell is observed (top of kymograph), as determined by the horizontal line formed by the leading edge. In contrast, the trailing edge (bottom of kymograph) undergoes spreading as can be observed by a distinct increase in cytoplasm at the back of the cell. In addition, the nucleus is no longer confined to the trailing edge of the cell. The cell now appears to have the symmetric morphology observed in Figure 2. In a majority of centrosome irradiated cells, random movement of the nucleus around the cell is observed as compared to control irradiated cells where no movement of the nucleus is observed (Movie S1 and S2). Over a 20 hour observation period, recovery of the polarized morphology did not occur in any of the centrosome irradiated cells. Cells irradiated within the cytoplasm (Figure 3B) maintained a polarized cell shape throughout the observation period with the nucleus located at the trailing edge of the cell (maximum 20 hours).

### Centrosome irradiation of wound-edge PtK2 cells

**Figure 4:**
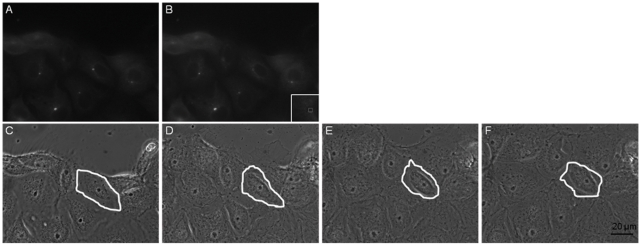
Centrosome irradiation of a wound edge PtK2 cell. Image (A) represents the GFP-centrin fluorescence signal prior to laser exposure. The area within the white box visible in the inset displays the ROI of laser exposure. Image (B) shows the GFP fluorescence signal following centrosome irradiation of a wound edge PtK2 cell. Images (C–F) are phase contrast images following the cell for 4.5 hours after the irradiation event: (C) immediately following irradiation, (D) 1.5 hours following irradiation, (E) 3 hours following irradiation, (F) 4.5 hours following irradiation. Over time, the centrosome irradiated cell (outlined in white) fails to migrate into the wound space. Neighboring control cells continue to migrate resulting in the irradiated cell falling behind the wound edge. Scale bar  = 20 µm. See Movie S3.

A similar change in cell morphology was observed in centrosome-irradiated PtK2 cells that were located at the edge of an induced wound in monolayer cultures (Figure 4 and Movie S3). Immediately following ablation, centrosome irradiated cells were capable of forming a distinct lamellae, not always in the direction of the wound as was the case for control cells. In most cases, centrosome irradiated cells migrated at a sufficiently slower rate than the control non-irradiated cells which were actively migrating towards the open wound area, as is typical in this wound-healing model. This resulted in the centrosome irradiated cell losing its position at the wound edge (Figure 4).

### Centrosome irradiation effect on migration

**Figure 5:**
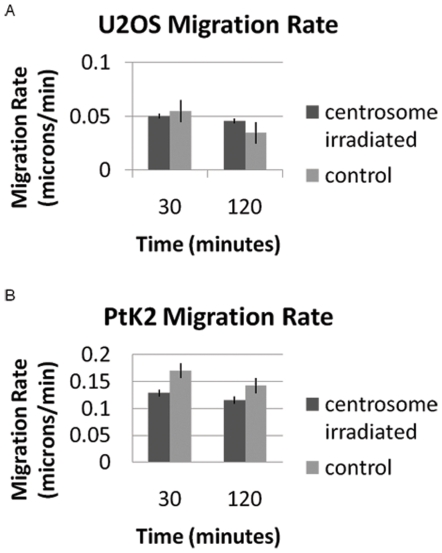
Centrosome irradiation results in a decreased rate of migration for U2OS and PtK2 cells. During a 2 hour observation period following irradiation, centrosome targeted U2OS cells migrated at a 61–69% slower rate when compared to control cells. U2OS measurements are based on 9 centrosome irradiated cells and 8 control cells. Centrosome targeted PtK2 cells migrated at a 19–24% slower rate when compared to control cells. PtK2 measurements are based on 10 centrosome irradiated cells and 18 control cells.

The rate of migration was calculated for U2OS and PtK2 cells 30 minutes and 2 hours following laser irradiation (Figure 5). PtK2 cells migrated at an average rate of 0.13 microns/min when observed thirty minutes post centrosome irradiation. Control cells migrated at an average 0.17 microns/min indicating a 24% decrease in migration rate with centrosome deletion (p = 0.16). At 2 hours post irradiation centrosome irradiated cells (n = 10) migrated at an average rate of 0.12 microns/min while control cells (n = 18) migrated at 0.14 microns/min (p = 0.36). Although not statistically significant, we observe a distinct trend of a slower migration rates for irradiated cells in comparison to neighboring control cells. Control cells always extended lamellae in the direction of the wound, while centrosome ablated cells formed a lamellae in a random direction, usually not toward the wound.

Interestingly, the U2OS cells did not display any significant change in migration rates when comparing centrosome-irradiated to control cells. At 30 minutes, control cells migrated at a rate of 0.06 microns/min and centrosome-irradiated cells at 0.05 microns/min. At 120 minutes, centrosome irradiated cells (n = 9) migrated at a slightly faster rate of 0.046 microns/min when compared to control cells (n = 8) that migrated at 0.035 microns/min.

### Centrosome irradiation effect on cell morphology

**Figure 6:**
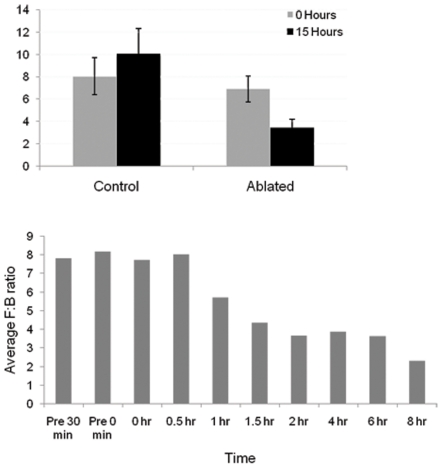
The F:B ratio quantifies the transition from a polarized to nonpolarized cell morphology occurring 30 to 90 minutes following centrosome irradiation. (A) A significant decrease in the average F:B ratio was observed for centrosome irradiated cells measured 15 hours following irradiation (p≤0.05). A statistically significant difference was observed between the 15 hour irradiated cells and both the control (cytoplasm irradiated) 15 hour and the zero hour centrosome irradiated. No difference was observed between the zero and 15 hour control. Error bars represent the calculated s.e.m. for each data set: control zero hour (1.7), irradiated zero hour (1.2), control 15 hour (2.3), irradiated 15 hour (0.8). (B) Changes in F:B ratio over time correspond with changes in cell shape between 30 minutes and 2 hours following irradiation. Chart shows a dramatic change in F:B ratio at 30 minute or 2 hour intervals beginning 30 minutes prior to irradiation to 8 hours following irradiation. A significant difference is observed for time points 1.5 hours and longer was observed (p≤0.05) as compared to the F:B value 30 minutes prior to irradiation. Significant p values are denoted by *. A total of 26 cells were observed for a minimum of 2 hours, 15 of which were followed for 4 hours and 11 followed for 8 hours.

Changes in cell morphology were quantified by the F:B ratio, as described in Material and Methods (Figure 6A). This ratio was quantified at two time points: immediately and 15 hours following irradiation. The ratio was calculated for 40 control and 40 centrosome-irradiated cells. A significant difference p<0.05 in the F:B ratio was observed when comparing immediately irradiated cells (both control and centrosome irradiated) to centrosome irradiated cells at the 15 hour time point. A significant difference between centrosome irradiated and cytoplasm irradiated cells was seen at the 15 hour time point. No significant difference was observed when compairing control cells at the two time points, or when comparing centrosome and cytoplasm irradiated cells immediately following ablation. Comparison of the F:B ratio demonstrates that between times t = 0 and t = 15 hours following irradiation of the centrosome, a significant change in cell morphology occurs. This change is not observed either immediately after irradiation or in cytoplasm irradiated cells.

To characterize the time scale of the change in cell morphology, images were acquired at 5 minute intervals following centrosome irradiation in 26 cells (Figure 6B). The F:B at 30 minutes prior to irradiation was 7.8, a baseline value to which subsequent time points were compared. F:B just prior to, post, and 30 minutes post ablation were 8.2(p = 0.87), 7.7 (p = 0.96) and 8.0 (p = 0.92) respectively. Between 30 minutes and 2 hours post ablation, a decrease in F:B ratio from 8.0 to 3.6 (p = 0.02) was observed. A decreasing trend of p values at 1 hr (p = 0.30), 1.5 hours (p = 0.05), and 2 hours (p = 0.02) was determined. Later time points 2 hr (p = 0.02), 4 hr (p = 0.05), 6 hr (p = 0.03), and 8 hr (p = 0.003) were significantly different than baseline. All cells were followed for a minimum of 2 hours. Fifteen cells were followed for 4 hours and 11 cells were followed for 8 hours. The data demonstrate a statistically significant change in cell morphology between 30 minutes and 2 hours following laser irradiation of the centrosome.

### Centrosome irradiation effect on microtubules

**Figure 7:**
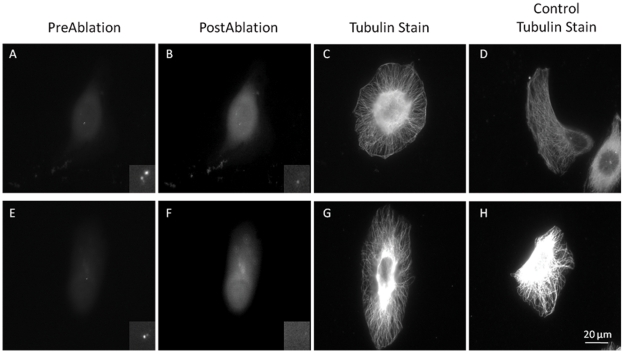
Centrosome irradiation causes a change in microtubule organization. Images A,E show GFP fluorescence prior to laser irradiation. Images B,F are immediately following laser exposure. Inset shows a magnified image of the GFP fluorescent centrosome region. Images C,D,G,H show cells fixed 2 or more hours following irradiation, and stained for B-tubulin. Cells with similar morphology prior to irradiation were matched horizontally. Cells with an irradiated centrosome display a nonpolarized microtubule network, unlike cytoplasm irradiated cells. Scale bar  = 20 µm.

The irradiation of the centrosome results in changes in microtubule organization over a 2 hour period. Figure 7 shows 2 examples of centrosome irradiated and 2 control cytoplasm-irradiated cells Fluorescence images from before (Figure 7A,E) and after irradiation (Figure 7B,F) are presented. The insets are computer-magnified images of the centrosome targeted region. Tubulin immunofluorescence images of the irradiated cells are shown in Figure 7C,G. Microtubules are evenly distributed throughout centrosome irradiated cells, whereas in cytoplasm-irradiated cells, a concentration of microtubules is observed between the nucleus and leading edge of the cells, as is expected for migrating cells. In addition to the change in the symmetric organization of the microtubules, microtubules were often observed encircling the periphery of the cell in the centrosome irradiated cells (Figure 7C) 2 hours following laser irradiation.

Cells fixed immediately following laser irradiation of the centrosome and labeled by tubulin immunofluorescence displayed a polarized microtubule network similar to the microtubule organization of control cells (Figure S2). Irradiated cells appeared to retain a polarized cell shape and microtubule network for up to 30 minutes following irradiation, which agrees with our measured of F:B ratio. No visible difference was observed between centrosome and cytoplasm-irradiated cells that were fixed and stained immediately following laser exposure.

### Quantification of changes in microtubule organization

**Figure 8:**
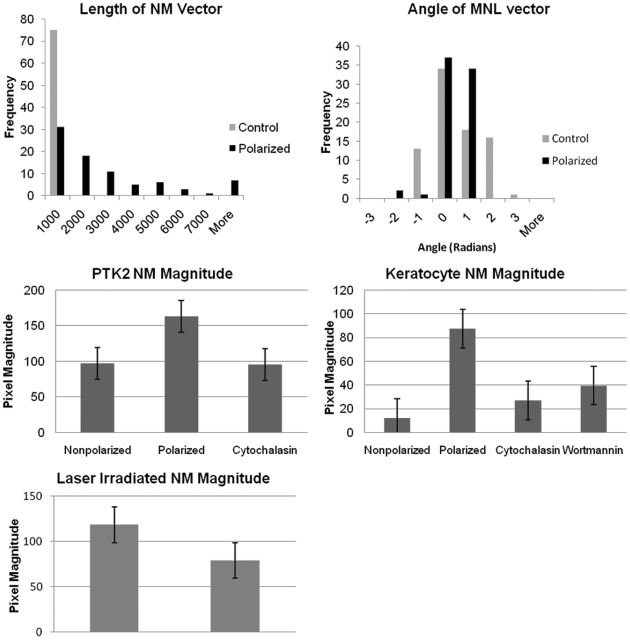
Quantification method to determine the polarization of the microtubule network. (A) A significant diference in NM magnitude was observed between nonpolarized and polarized PtK2 cells (p = 0.001). The increase in vector length reflects an asymmetric shift in microtubule organization in response to the scratch assay. (B) The angle between MN and NL vectors tightens about zero indicating alignment of NM along the direction of migration (NL). NM magnitude is significantly different between nonpolarized and polarized cells for both PtK2 and keratocytes. The NM magnitude was compared for two cell types: (C) PtK2 and (D) Keratocytes. Nonpolarized and polarized cells possessed a significantly different NM magnitude for both non-treated PtK2 (p = 0.003) and Keratocytes (p = 0.005). When polarized cells were treated with cytochalasin (actin destabilization) and wortmannin (PI3K inhibition), a significant difference for both PtK2 (cytochalasin p = 0.008) and Keratocytes (cytochalasin p = 0.002 and wortmannin p = 0.01) was observed. (E) A significant decrease in NM magnitude was seen when comparing average values for cytoplasmic irradiated cells (control n = 28) with centrosome irradiated cells (n = 27) analyzed 15 hours after laser exposure (p = 0.01). S.e.m. bars are displayed for each NM magnitude condition.

A dramatic reorganization of the microtubule network was observed within 2 hours following centrosome irradiation. Analysis of the changes in microtubule organization was performed by calculating the NM (nucleus-to-microtubule tip centroid) vector and NML (microtubule tip centroid-nucleus-lamellae) angle (see Materials and Methods section). This quantification method was initially tested on a confluent layer of PtK2 cells. A scratch in the cell monolayer induced directed migration and polarized microtubule networks in cells adjacent to the open scratch zone (the region devoid of cells). Cells were analyzed 2 hours following the induction of the scratch. As a control, cells from a different induced scratch were immediately fixed. Eighty-two cells at the two hour time point and 75 control cells were analyzed (Figure 8A,B). All control cells had a NM vector length of less than 2000 while 44 of 75 polarized cells had an NM vector of 2000 or greater. A statistically significant increase in vector length (p<0.05) reflected microtubule organization toward the lamellae (Figure 8A). In addition, a tightening of the NML angle distribution about zero radians, the direction of migration, was observed (Figure 8B). These changes represent a statistically significant asymmetric shift in the microtubule network towards the direction of migration and offer a quantifiable measure of polarization.

Control PtK2 cells and PtK2 cells treated with Cytochalasin-D were also analyzed following fixation with a scratch assay (Figure 8C). As expected, disruption of the actin network leads to an average NM magnitude similar to that of non-polarized cells observed in the previous scratch test (n = 36). A significant difference (p<0.05) between cytochalasin treated cells (n = 14) and control (non-treated) polarized cells (n = 39) was observed. In addition, the quantification method was tested on control and keratocytes treated with cytochalasin-D and wortmannin (PI3K inhibitor) (Figure 8D). Since keratocytes are small, fast migrating cells, their cell morphology was very different from those of larger, slower moving cells. A significant difference (p<0.05) between non-polarized, non-treated (n = 3) and polarized, non-treated keratocytes (n = 10) was observed. The number of non-polarized keratocytes analyzed was very small due to their natural polarized state. Both cytochalasin-D (n = 27) and wortmannin (n = 10) treated cells had smaller NM magnitudes similar to those of non-polarized cells. “P” values were significantly different from the polarized, non-treated keratocytes (p<0.05). Our results demonstrate that the microtubule quantification method based on the shift in NM magnitude was robust enough to analyze multiple cell types under cytoskeleton destabilizing conditions.

This microtubule analysis method was used to compare centrosome irradiated and cytoplasm irradiated U2OS cells fixed 15 hours following irradiation with subsequent tubulin immuno-staining (Figure 8E). A significant decrease in NM magnitude (p = 0.01) between cytoplasm irradiated (n = 28) and centrosome irradiated (n = 27) cells was observed. Using this method, a significant depolarization of the microtubule network following centrosome irradiation was observed and quantified.

### Centrosome irradiation effect on actin network

**Figure 9:**
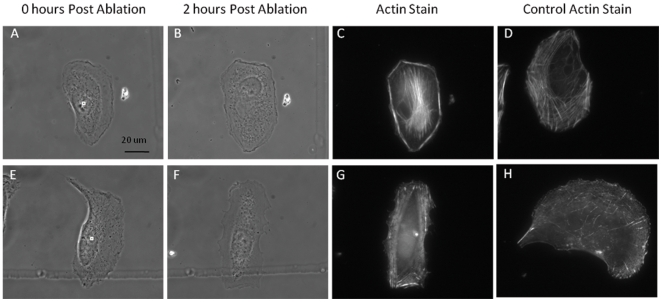
Centrosome irradiation causes a change in actin organization. Images A,E show the phase contrast image immediately (one second) after laser exposure. Images B,Fshow the phase contrast image after fixation 2 or more hours following laser exposure. Images C,D,H,I show the fluorescence images of cells stained with phalloidin. All images are the same magnification. Cells with similar morphology prior to irradiation were matched horizontally. Scale bar  = 20 µm.

The irradiation of the centrosome results in changes in the organization of the actin network over a 2 hour period (Figure 9). Images from figure 9A,E are phase contrast images immediately following centrosome irradiation. Images from figure 9B,F correspond to phase contrast images taken 2 hours following laser exposure. The change in cell morphology from highly polarized to non-polarized is visible by comparing the first 2 columns. Images from figure 9C,D,H,I correspond to phase images taken following fixation and immunofluorescence staining for actin. Cytoplasm irradiated (control) cells were matched to centrosome irradiated cells with similar cell morphology prior to laser exposure using phase images. The results of the actin staining suggest a dramatic change in actin organization. Many of the centrosome irradiated cells, especially those with actin bundles, lost the actin pool characteristic of the lamellae at the cell's leading edge. In addition, irradiation did not inhibit membrane ruffling (Movie S1). In contrast to the non-polarized centrosome irradiated cells, all control cells exhibited an asymmetric concentration of actin filaments at the leading edge indicative of a migrating phenotype.

## Discussion

Our experiments show that selective laser micro-ablation of the centrosome leads to qualitative and quantitative changes in the cell's ability to polarize. Interestingly, photoablation of a minute volume of the cell caused no obvious structural changes immediately following laser ablation. However, within the time scale of cytoskeletal remodeling, a significant difference between cytoplasm and centrosome ablated cells were observed. Resulting modifications indicative of changes in cell polarization included alterations to the overall cell morphology (F:B ratio), nuclear position, and microtubule and actin network reorganization.

There have been several previous studies in which laser irradiation has been used to damage or ablate centrosomes and/or centrioles with short-pulsed nanosecond lasers [16], [18], [19], [23]. The most relevant to this study was the Koonce et al. study in which a UV nanosecond pulsed laser was used to microirradiate the centrosome in migrating newt eosinophils. Following the irradiation of the centrosome region the cells lost their ability to migrate in a straight line and had a decreased migration rate. However, this study preceded the discovery of fluorescent fusion proteins and laser targeting relied on approximation of centrosome location based on phase contrast imaging. TEM analysis confirmed ablation of the centrosome, but did not describe the extent of nonspecific damage. Here, we used fluorescent fusion-protein labeled centrosomes, which allowed more precise targeting. In addition, we used an ultrashort 200 femtosecond pulsed laser whose effects are known to be confined to the laser focus spot.

We used an 800 nm NIR femtosecond laser for microirradiation, a system that was used previously to ablate single microtubules [22]. Organelle ablation by the femtosecond irradiation is based on nonlinear multiphoton interactions [24] and therefore only affect molecules at the laser focus and, when properly applied, offer no significant mechanical nor thermal perturbation of the cell. This improvement in targeting allows selective targeting of the centrosome thereby mitigating effects due to off-target ablation.

The goal of the study reported here is to understand the role of the centrosome in cell polarization and migration. We were able to monitor the loss of polarization as ablated cells reorganized into a symmetric morphology, an event that occurred between 30 and 90 minutes following laser irradiation. The change in cell morphology was accompanied by a change in the position of the nucleus and the rearrangement of the tubulin and actin cytoskeleton. Since the loss in cell polarization is not immediate, as would be expected if the centrosome played a role in maintaining mechanical stability, our results define a dual role for the centrosome in migrating interphase cells as that of a signaling nexus as well as a mechanical fulcrum.

We observed a decrease in the rate of migration for centrosome-irradiated wound-edge PtK2 cells as compared to non irradiated cells. Interestingly, no affect was observed in the migration rate for irradiated single U2OS cells. This difference can be explained by the fundamentally different environments of each cell type. The wounding of an epithelial sheet of cells, as in the PtK2 cells, causes a release of many proteins and signaling molecules, whereas the stimulation of single migrating U2OS cells were based solely on BMP2 stimulation. This suggests the centrosome plays a complex role in migration through signaling.

The change from a polarized to non-polarized state was characterized by the rear of the cell spreading, with respect to the nucleus, until it appeared indistinguishable from the lamellae. The changes in the back and sides of the cell likely involve changes in signaling molecules, cytoskeletal organization (such as actin pooling), and membranous structures (i.e. formation of focal adhesions). Following irradiation, lamellae-like protrusions at the rear and sides of the cell fused with the ‘true’ lamellae to form uniform, symmetric cell morphology. We can conclude that deletion of the centrosome does not preclude the cell from forming new focal adhesions and rearranging cytoskeletal components. It is likely that these processes are required to form and stabilize the observed lamellar protrusions and cell spreading.

Following the irradiation event, ruffling of the membrane at the cell periphery persisted indicating the constant addition of actin monomers to form filaments at the cell edge [7]. Thus the loss of the centrosome does not affect actin monomer accumulation and actin filament elongation at the cell periphery. However, changes in the actin network may result indirectly from changes in cell morphology and polarization.

Unexpectedly, the centrosome irradiated cell can still reorganize its microtubules but not in an asymmetric orientation as required for migration. For large slow migrating cells such as the U2OS cells the microtubules are essential for stabilization of the polarized lamellae. Removal of the centrosome causes the cell to lose its ability to maintain its polarization, and thus, directional migration. The loss of directed migration in two different types of cells from widely different species (PtK2, marsupial long-nosed rat kangaroo kidney cells, and U2OS, human osteosarcoma cells) suggests that the role of the centrosome in polarization may be conserved.

A previous study described a subset of microtubules that are derived from the trans Golgi network that preferentially orient toward the leading edge of motile cells [23]. We find that following centrosome irradiation, the microtubule network is organized in a radial array. If all of the centrosome derived microtubules depolymerized, then we would expect to see only the radial array of Golgi-derived microtubules remaining when analyzed using immunofluorescence. However, we see the opposite effect: microtubule network mimics only the centrosome-derived microtubules. These results suggest that the Golgi-derived microtubules are not sufficient to maintain polarization, and that the centrosome is required for preservation of polarized cell morphology.

Laser microirradiation permits the precise removal/ablation of a submicron region of the cell such as the centrosome or a region in the cytoplasm of equivalent size. An advantage of laser microirradiation is that it can produce controlled sub-micron focal ablations that do not result in wide scale perturbations like those observed following conventional drug treatments or radiation exposure. The immediate and precise ablation of the centrosome results in three possible outcomes: (1) no response, (2) rapid reorganization of the cytoskeleton leading to a change in cell morphology and polarization, and (3) a longer time period for reorganization of the cytoskeleton and the appearance of focal adhesions. Time lapse imaging of the irradiated cells suggests the third situation, a post-laser time period where reorganization of the cytoskeleton as well as focal adhesions occurs. In summary, our results demonstrate that removal of the centrosome results in loss of ability to maintain polarized cell morphology in two widely different cell models, migrating individual U2OS cells and PtK2 cells moving as part of a confluent sheet of cells. Significant changes in cell shape correspond to significant changes in the organization of the microtubule and actin networks occurring between 30 to 90 minutes following centrosome ablation. These results demonstrate that the centrosome plays a key role in to maintenance polarization during the cell migration process.

## Materials and Methods

### Cell Culture

Human osteosarcoma cells (U2OS) expressing GFP centrin were graciously provided by Christine Suetterlin (Department of Developmental and Cell Biology, University of California, Irvine). Cells were cultured in advanced DMEM media (Gibco) supplemented with 2% FBS. Cells were plated 24 hours prior to experiments on glass bottom dishes (Mattek) at a very low density (approximately 5–10% confluency) to allow for a maximum amount of individual cells (cells not in contact with other cells). Prior to imaging, DMEM media was replaced with L-15 media buffered with HEPES and supplemented with 10% fetal bovine serum (FBS). Migration was stimulated by the addition of bone morphogenic protein-2 (BMP-2) (Sigma) into the media at a final concentration of 0.1 µg/mL.

*Potorous tridactylis* kidney epithelial (PtK2) cells were cultured as previously described (Wakida et al., 2007). Cells were plated 24 to 72 hours prior to imaging. Media was also replaced with L-15 media buffered with HEPES as previously described. Once cells reached 100% confluency, a wound was created in the cell monolayer using a plastic pipette tip. Wound-edge cells were targeted for laser irradiation and/or followed during experiments.

During imaging experiments, PtK2 and U2OS samples were kept at 37°C using a Warner Instruments (Hamden, CT) TC344B temperature controller.

Goldfish keratocytes were acquired from fish scales placed on glass bottom dishes filled with L-15 media. A sterile glass coverslip was placed on top of the scales to assist in attachment to the dish surface. Keratocytes were stored at room temperature for 24–48 hours prior to imaging.

Cytochalasin and wortmannin were used to destabilize the actin network and inhibit PI3Kinase localization. Wortmannin (Sigma) was used to inhibit Phosphatidylinositol-3-kinases at a 10 nM concentration. Cytochalasin D was used at 1 ng/mL of media.

### Immunofluorescence

Cells were fixed at designated time points with 4% paraformaldehyde for 10 minutes. Fixative was then replaced with a 0.1% Triton × and 2.5% fetal bovine serum blocking solution. Following an overnight incubation in blocking buffer, cells were treated with a 1∶1000 dilution (1∶100 for pericentrin) of primary antibody to blocking buffer for 1 hour. Polyclonal rabbit anti pericentrin and anti ninein antibodies (Abcam) as well as monoclonal mouse anti gamma tubulin (Sigma) were used as centrosomal markers for immunofluorescence studies. A monoclonal mouse anti β-tubulin antibody (Sigma) was used for visualization of the microtubule cytoskeleton. Cells were washed three times in PBS for a 5 minute period. Cells were then incubated with a 1∶500 ratio of secondary antibody for one hour. 2 µg of Alexa Fluor 430 goat anti rabbit and Alexa Fluor 594 goat anti mouse were used per sample. Following staining, cells were re-located by comparing patterns of the cells to phase images acquired prior to fixation (Figure 1E,J,O).

### Laser and Microscope Setup

A Coherent Mira 900 Ti:sapphire laser (Coherent Inc., Santa Clara, Ca) operating at 780 nm and emitting 200 femtosecond pulses at 76 MHz was used for irradiation. The beam was focused into a dual camera adaptor affixed to the left port of a Zeiss Axiovert 200M inverted microscope. The dual camera adaptor includes a dichroic mirror reflecting infrared light and passing visible light. The beam was directed into a Zeiss 1.4NA 63× oil immersion apochromat objective lens. Laser power was controlled by a polarizer mounted in a motorized rotational stage. Laser exposure was controlled electronically by a Uniblitz shutter controller (see Figure S3 for optical set-up).

Laser exposure time was set at 50 ms (3.8×10^6^ femtosecond laser pulses) as determined to be the minimum time for consistent irradiation results at the reported peak irradiance. For all experiments, laser power after the objective was 30 mW (irradiance 5.5×10^11^ W/cm^2^, energy deposited per pulse 4.2×10^9^ J/cm^2^). The position of the beam was controlled by a Newport FSM-300 series fast scanning mirror (Newport Corp., Irvine, CA). As controlled by the custom “Robolase” software [25], the fast scanning mirror allows for the user to specify any region of interest (ROI) for laser irradiation. Robolase calculates the number and position of pulses required to fill the specified ROI and automatically exposes each position to the laser. A Zeiss Axiovert 200M with a motorized focus allows for 3 planes in the z-axis to be exposed to the laser.

Images were acquired using a Hamamatsu Orca-ER CCD Camera attached to a dual camera adaptor at the left port. A fluorescence illumination system Exfo Xcite 120 lamp (Canada) was used as a light source for fluorescence imaging. Exposure of the sample to the excitation light source was controlled by a second Uniblitz shutter and controller.

### Quantification of migration rate

To quantify the migration rate of U2OS and PtK2 cells, cells were manually outlined using ImageJ software. The software calculated the x and y value of the centroid. Migration rates were determined by the distance (in microns) between the calculated centroid and its initial position following irradiation, divided by the minutes of observation from laser exposure. Student's t test was used to determine statistcal significance between groups for all data analysis.

### Quantification of cell shape

**Figure 10:**
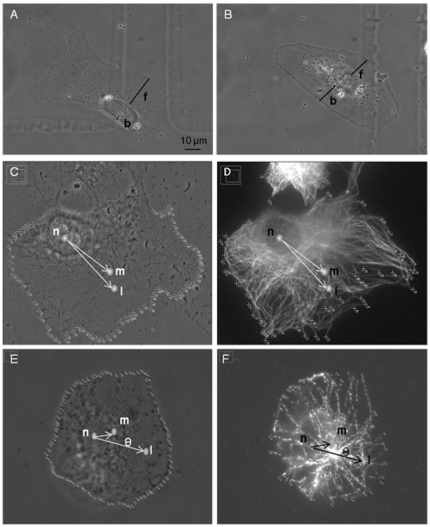
Quantification of cell shape and microtubule network. (A and B) Quantification of polarized or nonpolarized cell morphology by phase contrast imaging. The shortest distance between the nucleus edge and the lamellae, F, and nucleus edge and trailing edge, B, were calculated. Since polarized cells (A) possess a large lamellae, the ratio of F:B was expected to be greater than 1. For nonpolarized cells (B), the F:B ratio is expected to be approximately 1. (C–F) Quantification of a polarized vs. nonpolarized microtubule network. Three points are used to quantify the state of the cell: N (nucleus centroid), M (microtubule tip centroid), and L (lamellae). (C and D) show the microtubule network of a polarized cell. In the case of polarized cells, NM magnitude is large and MNLθ is small corresponding to asymmetry of the microtubules with respect to the direction of the lamellae. (E and F) show the organization of a nonpolarized cell with a larger value for MNL θ and a small magnitude for NM. Scale bar  = 10 µm.

To quantify changes in polarized cell shape, the shortest distance between the cells' nucleus edge and both the lamellae (front) and trailing edge (back) were calculated (F:B ratio). Measurements were based on phase contrast images from both live and fixed cells (Figure 10A,B). Polarized cells were expected to display a F:B ratio greater than one, while non-polarized cells were expected to display a ratio around 1.

### Quantification of microtubule cytoskeleton

To quantify the polarization state of the microtubule network, both phase contrast (Figure 10C,E) and fluorescent images (Figure 10D, F) from cells stained for tubulin were analyzed. First the pixel coordinates of all visible microtubule tips at the periphery of the cell were determined. The mean position of all of the microtubule tips was used as the microtubule tip centroid, M. Next, the pixel coordinates of both the lamellae (L) and nucleus centroid (N) were found. Using these three coordinates, it was possible to compare the asymmetric shift of the microtubules by comparing the length of the NM vector (pointing from N to M and determining the angle between the NM vector and the direction of migration (L) determined by the NL vector (pointing from N to L), or NML θ. A shift in the direction of migration would decrease the value of nml θ with respect to zero radians. The location of the 3 calculated positions (n, m, l) are illustrated for a polarized cell (Figure 10C, D) and a non-polarized cell (Figure 3E,F). The pixel positions used to calculate l is shown in Figure 8C and E. The pixel positions used to calculate m are displayed on Figure 10D and F.

## Supporting Information

Figure S1 Cytoplasm irradiated cells retain polarized cell morphology. A random region in the cytoplasm was irradiated at the same settings and the same area (black box in image B) as for centrosome irradiation. Images A,D,G,J are phase contrast images from the beginning of the observation period, 30 minutes prior to laser irradiation. Images B,E,H,K are taken immediately after irradiation. Images in C,F,I,L images taken 90 minutes or more after irradiation. A total of 15 cytoplasm irradiated cells were observed. Scale bar  = 10 µm.
(TIF)

Figure S2 Immunofluorescence staining of tubulin for 2 centrosome irradiated cells fixed immediately following laser irradiation. (A and D) are fluorescent images of GFP-centrin labeled U2OS cells before irradiation. (B and E) are fluorescent images taken immediately after irradiation. Green boxes depict irradiated ROIs. Images (C and F) show cells stained for tubulin. Cells show no collapse in the microtubule network following centrosome irradiation. Scale bar  = 10 µm.
(TIF)

Figure S3 Schematic diagram of the femtosecond laser irradiation and imaging system. The beam of a Coherent Ti:Sapphire laser is directed through a motorized shutter, polarizer, beam expander, fast steering mirror and an external lens before entering a dual camera adaptor fixed to a Zeiss Axiovert inverted microscope. Laser power measurements indicated 62% transmission (of total power at the laser head) at position a, 35% transmission before entering the objective, and 19% transmission at focal plane of objective.
(TIF)

Movie S1 Loss of polarized cell morphology following laser irradiation of the centrosome in U2OS cells stably expressing GFP centrin. Images acquired using time lapse phase contrast microscopy. Frames were acquired every 5 minutes for 19.25 hours.
(MP4)

Movie S2 Maintenance of polarized cell morphology following laser irradiation of the cytoplasm in U2OS cells stably expressing GFP centrin. Images acquired using time lapse phase contrast microscopy. Frames were acquired every 5 minutes for 10.8 hours.
(MP4)

Movie S3 Centrosome irradiated wound edge cell falls behind neighboring control cells as they migrate into the wound. Centrosome irradiated cell outlined in white on first frame. PtK2 cells stably expressing YFP centrin-2. Images were acquired using time lapse phase contrast microscopy. Frames were acquired every 10 minutes for 10.6 hours.
(MOV)
