# Supplementary figures and images for: An Intact Centrosome Is Required for the Maintenance of Polarization during Directional Cell Migration
Source: PLoS One. 2010 Dec 23;5(12):e15462. doi: 10.1371/journal.pone.0015462 (PMC3009746; doi:10.1371/journal.pone.0015462)

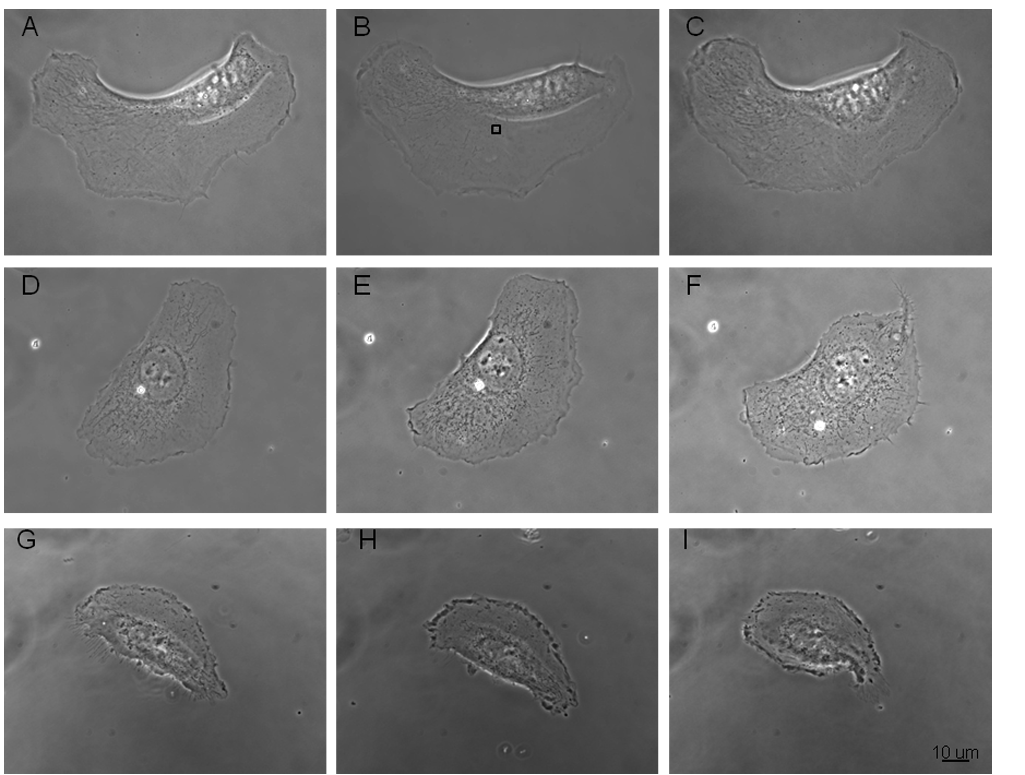

Supplement: Figure S1 — Cytoplasm irradiated cells retain polarized cell morphology. A random region in the cytoplasm was irradiated at the same settings and the same area (black box in image B) as for centrosome irradiation. Images A,D,G,J are phase contrast images from the beginning of the observation period, 30 minutes prior to laser irradiation. Images B,E,H,K are taken immediately after irradiation. Images in C,F,I,L images taken 90 minutes or more after irradiation. A total of 15 cytoplasm irradiated cells were observed. Scale bar = 10 µm. (TIF) [file pone.0015462.s001.tif]

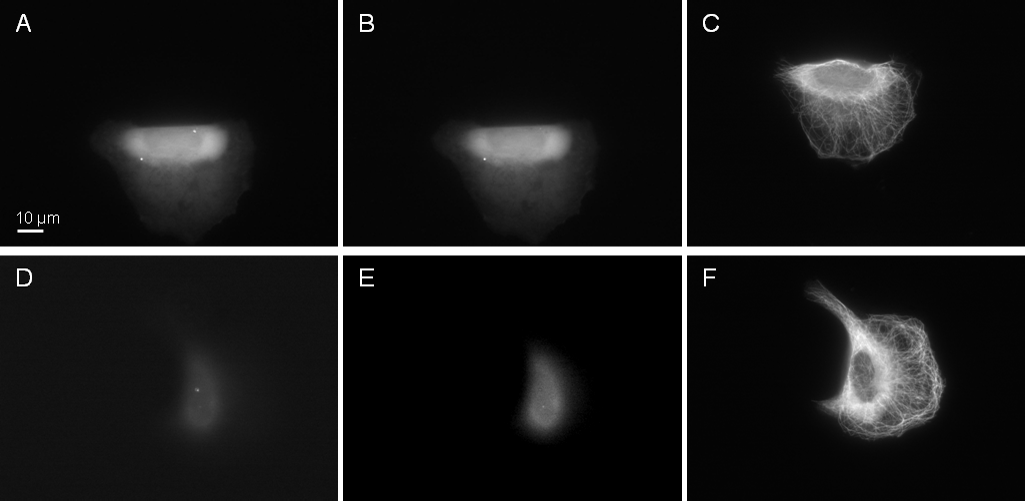

Supplement: Figure S2 — Immunofluorescence staining of tubulin for 2 centrosome irradiated cells fixed immediately following laser irradiation. (A and D) are fluorescent images of GFP-centrin labeled U2OS cells before irradiation. (B and E) are fluorescent images taken immediately after irradiation. Green boxes depict irradiated ROIs. Images (C and F) show cells stained for tubulin. Cells show no collapse in the microtubule network following centrosome irradiation. Scale bar = 10 µm. (TIF) [file pone.0015462.s002.tif]

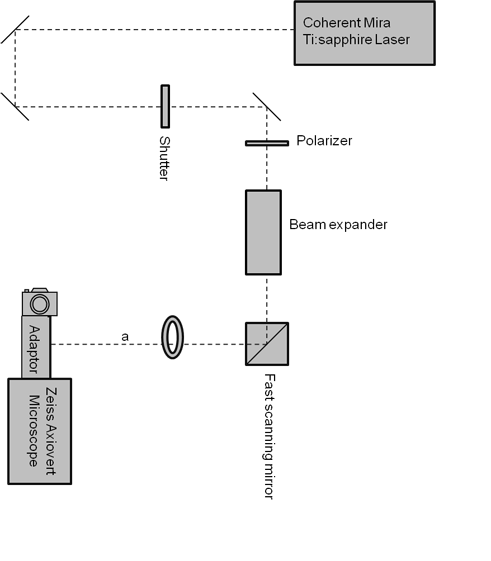

Supplement: Figure S3 — Schematic diagram of the femtosecond laser irradiation and imaging system. The beam of a Coherent Ti:Sapphire laser is directed through a motorized shutter, polarizer, beam expander, fast steering mirror and an external lens before entering a dual camera adaptor fixed to a Zeiss Axiovert inverted microscope. Laser power measurements indicated 62% transmission (of total power at the laser head) at position a, 35% transmission before entering the objective, and 19% transmission at focal plane of objective. (TIF) [file pone.0015462.s003.tif]
